# Replication-directed sister chromosome alignment in *Escherichia coli*

**DOI:** 10.1111/j.1365-2958.2009.06791.x

**Published:** 2009-07-24

**Authors:** Xun Liu, Xindan Wang, Rodrigo Reyes-Lamothe, David Sherratt

**Affiliations:** Department of Biochemistry, University of OxfordOxford OX1 3QU, UK

## Abstract

Non-replicating *Escherichia coli* chromosomes are organized as sausage-shaped structures with the left (L) and the right (R) chromosome arms (replichores) on opposite cell halves and the replication origin (*oriC*) close to midcell. The replication termination region (*ter*) therefore passes between the two outer edges of the nucleoid. Four alignment patterns of the two <LR> sister chromosomes within a cell have been detected in an asynchronous population, with the <LRLR> pattern predominating. We test the hypothesis that the minority <LRRL> and <RLLR> patterns arise because of pausing of DNA replication on the right and left replichores respectively. The data resulting from transient pausing or longer-term site-specific blocking of replication show that paused/blocked loci remain close to midcell and the normally replicated-segregated loci locate to the outer regions of the nucleoid, therefore providing experimental support for a direct mechanistic link between DNA replication and chromosome organization.

## Introduction

The intimate relationship between replication and chromosome segregation is a central feature of the *Escherichia coli* cell cycle. Synthesis of two daughter duplexes from each replication fork is followed by a variable cohesion period (Nielsen *et al*., 2006a; Espéli *et al*., 2008; Reyes-Lamothe *et al*., 2008; Wang *et al*., 2008), in which newly replicated loci are not spatially separable as a consequence of interwrapping of the newly replicated duplexes (pre-catenation). After removal of pre-catenanes by topoisomerase IV, newly replicated sister loci segregate to opposite cell halves (Wang *et al*., 2008). The replication origin-terminus axis divides the circular chromosome into the left (L) and the right (R) replichores, which locate to opposite halves of each sister chromosome in a growing cell and therefore to opposite cell halves in a newborn cell (Nielsen *et al*., 2006b; Wang *et al*., 2006). The predominant pattern of sister chromosome alignment (SCA) is <LRLR>, rather than the more intuitive <LRRL> or <RLLR> patterns that might be expected to arise in bilaterally symmetrical daughter cells. We proposed that the <LRLR> translational symmetry might arise if daughter replisomes moved towards the cell quarter positions after initiation of replication at midcell (Wang *et al*., 2006). Indeed this was subsequently demonstrated experimentally by showing that within 5 min of replication initiation sister replisomes migrate to opposite cell halves, where they appear to track along the DNA, eventually moving back to midcell at replication termination (Reyes-Lamothe *et al*., 2008). A subsequent study has demonstrated that in <LRLR> cells, the leading strand templates of the left and right forks are segregated to the outer nucleoid edges and the lagging strand templates are segregated internally within the nucleoid (White *et al*., 2008). Nevertheless, segregation of both leading strand templates to the outer nucleoid edges is not mandatory for segregation, as in cells with the minority <LRRL> and <RLLR> SCA patterns, an equal mixture of leading and lagging strand templates must be segregated to the outer nucleoid edges.

We also proposed that the minority SCA patterns, <LRRL> and <RLLR> (24%), arise when replication is paused or blocked on the replichore that is found internal to the sister nucleoids, close to midcell/the invaginating septum (Wang *et al*., 2006). As the pattern frequencies were the same in RecA^+^ and RecA^-^ strains, they do not arise as a consequence of recombinational repair. Replication fork stalling or breaking appears to occur in most generations and is likely to be responsible for the low viability of *priA* and *recA* strains. The frequency of chromosome dimer formation by homologous recombination, which is assumed to largely result from replication forks stalling or breakage, has been estimated to be ∼15% per cell generation and has been proposed to result from a minority of such recombination events (Steiner and Kuempel, 1998; Michel *et al*., 2000; Pérals *et al*., 2000). Furthermore, tightly bound repressors can arrest replication forks, with as few as three LacI-*lacO* complexes arresting the *E. coli* replication fork *in vitro*, while overexpression of LacI and TetR repressors results in strong replication blocks when binding tightly to *lacO* or *tetO* arrays *in vivo* (Possoz *et al*., 2006; Payne *et al*., 2006). We show that transient or longer-term site-specific replication pausing/blocking increases the proportion of cells with the <LRRL> and <RLLR> patterns, with the blocked locus/loci remaining close to midcell or the newly formed septum, thereby demonstrating a direct causal link between DNA replication and chromosome segregation choreography.

**Fig. 1:**
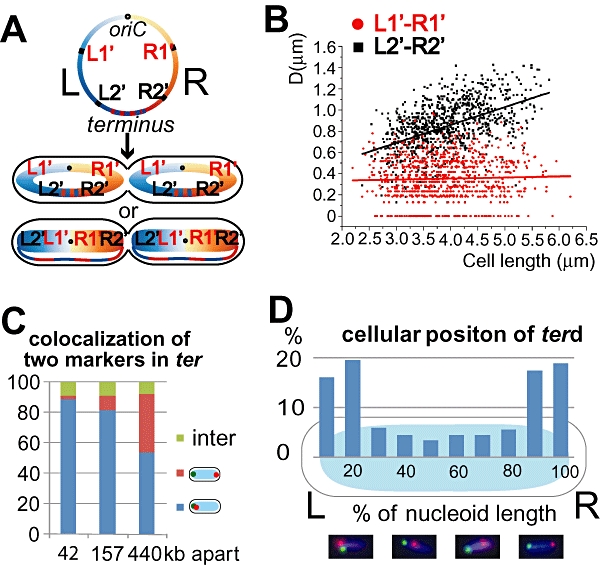
A. The doughnut and sausage models for *E. coli* chromosome organization. The genomic position of L1′, R1′, L2′ and R2′*parS* loci are shown on a simplified *E. coli* genome map (left panel), and their predicted cellular positions according to the two models are shown in two cartoon cells (right panel). The L and the R replichores are shown in blue and orange, respectively, on the genome map and in the schematic cells. Both schematic cells shown adopt a <LRLR> SCA. B. Average distances of L1′-R1′ and L2′-R2′ focus pairs plotted against cell length. In this plot, each D_L1′-R1′_ (or D_L2′-R2′_) represents the average of the two d_L1′-R1′_ (or d_L2′-R2′_) in each cell. The red (or black) line shows the linear best fit for all the R1′-R2′ (or L1′-L2′) data points. ∼1000 cells, or ∼2000 focus pairs, were analysed for each strain. C. Colocalization of two *ter* loci. Three strains with pairs of loci in *ter* separated by 42 kb (*ter*d and *ter3*), 157 kb (*ter2* and *ter3*) and 440 kb (*ter2* and *ter4*; see Wang *et al*., 2005; 2006) were examined. Young cells (cells with one focus for each locus and with no visible nucleoid splitting) were classified into three types: those with the two loci at the same pole (blue), at opposite poles (red) and at an intermediate position. Shown are the averages of two independent experiments for each strain, with more than 400 cells analysed for each experiment. D. Cellular positioning of a *ter*d locus (8 kb clockwise from *dif*) in young cells. The precise longitude position of the *ter*d locus within the nucleoid was measured in a strain colabelled with L3 for directionality. The population was binned into 10 groups according to the position relative to the nucleoid (illustrated as an oval) and the percentages are shown in histograms. A total of 288 cells were measured. Examples of individual micrographs are shown underneath, *ter*d marker in red, L3 in green.

The overall shape of the *E. coli* <LR> chromosome in the cell also requires clarification. Data from an earlier study suggested a ‘doughnut’ organization, which depicts the *E. coli* chromosome as a compacted ring structure with similar densities of DNA and compaction levels around the whole circle (Niki *et al*., 2000). Nevertheless, more recent data provide support for organization as a ‘sausage’, in which more than 75% of the chromosome is organized as a compacted rod, with the replication origin located close to midcell and the replication terminus region (*ter*) connecting the two outer nucleoid edges (Wang *et al*., 2006; Cui *et al*., 2007; Fig. 1A). We provide new evidence to support the sausage model of chromosome organization, with less than 50 kb of DNA being able to link the two outer nucleoid edges, and demonstrate that four SCA patterns (*cis*-<LRLR>, *trans*-<LRLR>, <LRRL> and <RLLR>) coexist and interconvert in a slow-growing *E. coli* population with a non-random distribution. Furthermore, we show that the SCA a cell adopts is independent of that of its mother in the previous cell cycle.

## Results

### Sausage or doughnut?

To distinguish between the doughnut and sausage models for chromosome organization (Fig. 1A), we constructed two strains that would allow us to visualize the L1′-R1′ or L2′-R2′ locus pairs in live cells. L1′ is 948 kb counterclockwise of *oriC*, while L2′ is 1833 kb counterclockwise of *oriC.* R1′ is 893 kb clockwise of *oriC*, while R2′ is 1708 kb clockwise of *oriC*. In these two strains, L1′ and L2′ loci were labelled by YFP-pMT1-ParB bound to an inserted pMT1*parS* site, while R1′ and R2′ loci were labelled by CFP-p1-ParB bound to an inserted p1*parS* site. Previous studies have shown that the binding of these ParB fusion protein molecules to their cognate *parS* sites cause no appreciable growth defects under the growth conditions used here, in which replication initiation normally occurs within the same cell as termination (Nielsen *et al*., 2006a,b;).

Longer cells with two L1′-R1′ (or L2′-R2′) focus pairs per cell were analysed because we were unable to be certain whether or not shorter cells with one L1′-R1′ (or one L2′-R2′) focus pair had ongoing replication upstream of the loci. If the doughnut model is correct, D_L1′-R1′_≥D_L2′-R2′_, with D_L1′-R1′_ being the average longitudinal distance between an L1′ focus and a R1′ focus in the same cell half. If the sausage model is correct, D_L1′-R1′_ < D_L2′-R2′_ (Fig. 1A). The cluster of D_L1′-R1′_ distances is clearly lower than the cluster of D_L2′-R2′_ distances (Fig. 1B). Furthermore, the mean of all D_L1′-R1′_ (0.35 μm) is significantly smaller than the mean of all D_L2′-R2′_ (0.84 μm). As the cell length distributions of these two strains are practically identical (mean cell length 3.9 μm; Fig. S1A), we conclude that on average D _L1′-R1′_ is significantly smaller than D_L2′-R2′_ in slow-growing *E. coli* cells, thereby lending further support to the sausage model.

A direct consequence of the sausage model is that *ter* DNA must link the outer nucleoid edges. The average amount of *ter* DNA that links the outer edges of the nucleoid remains unclear, although it could be as little as 5 kb if uncompacted. Although we have no data to suggest that the level of compaction or the nature of *ter* organization in the linker region is different from that of the bulk nucleoid, MatP binding to multiple *matS* sites facilitates organization and compaction of *ter* into an apparent macrodomain (Mercier *et al*., 2008). Whereas, these and other data suggest that *ter* constitutes a single macrodomain, in which a range of fluorescent genetic loci show a high level of colocalization (Espéli *et al*., 2008), our own experiments have shown that *ter* markers are frequently resolvable and can locate to opposite nucleoid edges (Wang *et al*., 2006; below).

We now show, using fluorescent repressor-operator system (FROS), that *ter* loci 42 kb apart are spatially resolvable and form clear foci like those elsewhere in the chromosome. Whereas such closely spaced loci normally colocalize to the same nucleoid edge, in a small fraction of young cells that have not initiated replication, or are early in their replication cycle, they localize to opposite nucleoid edges (Fig. 1C and 2.2%). Therefore, less than 50 kb can link the outer nucleoid edges, and it is possible that as little as 5 kb of uncompacted DNA may constitute the linker, at least in some cells. Not surprisingly, increased spacing between *ter* loci increases the probability that they will localize to opposite nucleoid edges (Fig. 1C).

In young cells that are expected to have a non-replicating or early replicating nucleoid, > 80% have a locus 8 kb clockwise of *dif* (*ter*d), located at the outer nucleoid edge, with < 10% of *ter*d loci located at the nucleoid centre and a further 10% between the nucleoid centre and its outer edges (Fig. 1D). Once replicated close to midcell, the sister *ter*d loci separate to form two closely spaced foci at midcell, with one then usually migrating to a distal pole, thereby generating the translational symmetry pattern (Wang *et al*., 2006). The *ter*d locus segregates with the left and right replichores with equal frequency (Fig. 1D). The data also show that the compacted DNA of a focus can occasionally be found in the middle of the linker region, although we cannot eliminate the possibility that repressor binding itself compacts DNA that would otherwise be uncompacted. We also cannot rule out the possibility that foci in the middle of the linker region represent DNA that is still segregating to a nucleoid edge. We have never observed cells that lack a strong *ter*d focus, or have a focus that appears to be dispersed across the linker region. Therefore, we suspect that the linker is compacted to a similar extent as other chromosomal DNA. We propose that any part of *ter* can act as the linker and potentially span the outer nucleoid edges, although only a small fraction of this acts as the linker in any given nucleoid. For example, a linker in the range of 20–80 kb, derived on average from any segment of *ter*, would be expected to yield a given locus being in the centre third of the nucleoid 3–7% of the time, similar to the observed frequency.

### Sister chromosome organization

**Fig. 2:**
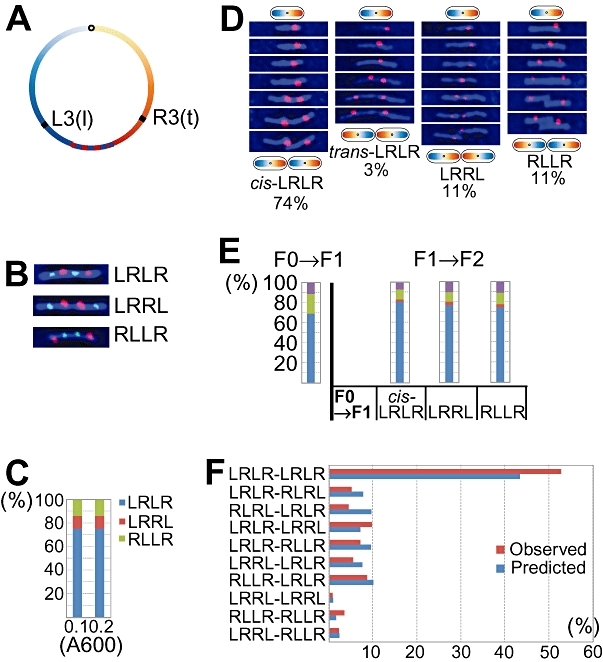
A. The genomic positions of L3 *lacO* and R3 *tetO* array loci. B. Snapshot images of cells adopting <LRLR>, <LRRL> or <RLLR> SCA. The L3 (or R3) foci are shown in green (or red). C. Proportions of cells with <LRLR> (blue), <LRRL> (red) or <RLLR> (green) SCA. Three experiments at different A_600_ are presented and ∼1000 cells were analysed for each experiment. D. Four time-lapse series (20 min interval) of F0→F1 cell generations adopting *cis*-<LRLR>, *trans*-<LRLR>, <LRRL> or <RLLR> SCA. The R3 foci are shown in red. The proportions of F0→F1 events adopting each SCA are shown at the bottom of each column. ∼3000 cell generations from 20 experiments were pooled and presented. E. Proportions of F0→F1 or F1→F2 events adopting *cis*-<LRLR> (blue), *trans*-<LRLR> (red), <LRRL> (green) or <RLLR> (purple) SCA. The first column shows the SCA proportions of the F0→F1 events (615 generations). The next three columns show the SCA proportions of the *cis*-<LRLR> (844 events), <LRRL> (240 events) or <RLLR> (142 events) F1→F2 groups. The *trans*-<LRLR> F1→F2 group was not included because it was too small (four events). F. Predicted and observed proportions of eight-focus (four sister chromosomes) cephalexin filaments. Predicted proportions are shown in blue (Supporting information) and observed proportions are shown in red.

To extend understanding of sister chromosome organization in live cells, we visualized the cellular localization of L3 and R3 loci, which were labelled by LacI-CFP bound to an inserted 240-repeat *lacO* array and by TetR-YFP bound to an inserted 240-repeat *tetO* array respectively (Fig. 2A; inserted 1655 kb anticlockwise of *oriC* and 1568 kb clockwise of *oriC* respectively). Using this FROS, L3 and R3 have been previously shown to locate near the two ends of the nucleoid (Wang *et al*., 2006), which make them good markers for the L and R replichores respectively. Saturating concentration of IPTG and anhydrotetracycline (AT) were present in the growth medium to reduce LacI and TetR binding, thereby minimising pausing or blocking of replication within the region of the array (Wang *et al*., 2005; Possoz *et al*., 2006).

Three SCAs were identified in snapshot pictures of the exponentially growing cell population (Fig. 2B; Wang *et al*., 2006). During early exponential phase (A_600_ = 0.1–0.2), <LRLR> was the major SCA adopted by 75% of the cells. <LRRL> and <RLLR> were two minor SCAs adopted by 10% and 14% of the cells respectively (Fig. 2C). In comparison, a previous FROS analysis on a smaller data set gave <LRLR> values in the range 76–88%, while a FISH analysis of 240 fixed wild-type cells gave proportions of <LRLR>, <LRRL> and <RLLR> of 84%, 8% and 8% respectively (Wang *et al*., 2006). When cells were grown on agarose gel pads containing growth medium with saturating amounts of IPTG and AT, only R3 TetR-YFP foci could be consistently visualized; L3 LacI-CFP foci were often indistinct and were therefore not scored (data not shown). Time-lapse tracking of R3 during ∼3000 F0 (mother)→F1 (daughter) events revealed that all four possible alignments for the two <LR> sister chromosomes arise in slow-growing *E. coli*: *cis*-<LRLR> (<LRLR> from a <LR> mother), *trans*-<LRLR> (<LRLR> from a <RL> mother), <LRRL> and <RLLR> (Fig. 2D). This extends our previous work, which has already shown that polar migrations of a newly replicated locus in a <LRLR> cell generate <LRLR> <LRLR> sisters in most generations (Wang *et al*., 2006).

There are two scenarios in which the proportions of the four SCAs could be propagated through generations. In scenario 1, a daughter cell generated by a mother cell that has adopted <LRRL> during the mother→daughter generation will only adopt <LRRL> during the daughter→granddaughter generation. In scenario 2, every cell has the same potential to adopt all four SCAs according to a common probability, determined by an unknown mechanism, regardless of the chromosome organization of its mother. To test these two scenarios, we extracted 615 F0 (mother)→F2 (granddaughter) events from the same body of data used to generate Fig. 2D (see Fig. S2A for two examples). We then sorted the 1230 F1 (daughter)→F2 (granddaughter) events into four groups according to the SCA their mothers adopted during the F0→F1 generation (Fig. 2E). If scenario 1 were correct, then the *cis*-<LRLR> F1→F2 group should be 100% *cis*-<LRLR>, the <LRRL> F1→F2 group should be 100% <LRRL> and the <RLLR> F1→F2 group should be 100% <RLLR>. However, the SCA proportions of the *cis*-<LRLR>, <LRRL> and <RLLR> F1→F2 groups are similar to each other and to the SCA proportions of the F0→F1 group (Fig. 2E), which is consistent with scenario 2.

To test how closely scenario 2 matches the propagation of SCA proportions from F0 to F2, cells were grown to A_600_∼0.1 and cell division was then inhibited with cephalexin (100 μg ml^−1^) for one generation. According to scenario 2, there should be 10 types of eight-focus cephalexin filaments and their proportions could be calculated using the SCA proportions of the F0→F1 events and of the F1→F2 events, both of which could be obtained from experimental data (see Supporting information for details). All the 10 types of eight-focus filaments were indeed identified in snapshot pictures (see Fig. S2B for examples), and their observed proportions broadly matched the predicted proportions (Fig. 2F). We conclude that scenario 2 closely reflects the real situation.

### Consequences of site-specific replication pausing and blocking

So what is the nature of the mechanism that commits a cell to one of four SCAs, with the <LRLR> translational symmetry of loci predominating? In particular, does the proposal that the switch from <LRLR> to <LRRL>, or <RLLR> arises when a replication fork stalls or pauses, with the paused or blocked locus generating the sister loci close to midcell (Wang *et al*., 2006)?

**Fig. 3:**
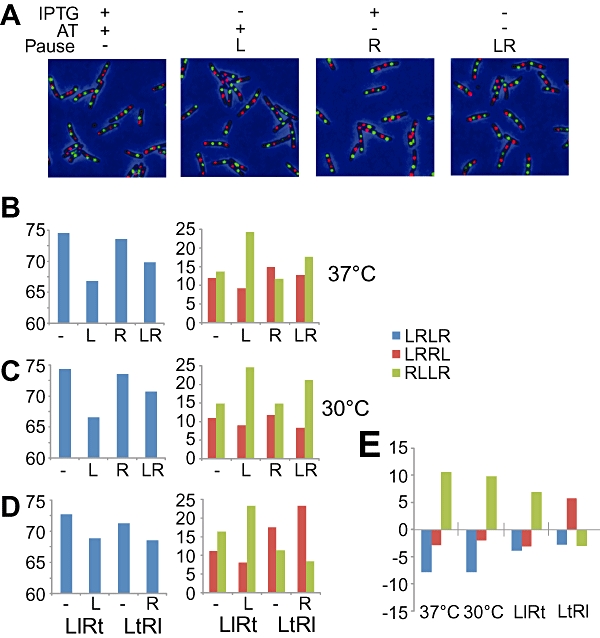
A. Snapshot images of cells growing in 37°C medium with the indicated combinations of inducers at saturating concentrations (IPTG: 0.5 mM, AT: 40 ng ml^−1^). ‘Pause’ refers to the replichore(s) affected by repressor-operator tight binding. The L3 foci are shown in green and the R3 foci in red. ∼2000 cells were analysed for each growth condition and the results are shown in Fig. 3B and C. B. The SCA proportions (*cis*-<LRLR> in blue, <LRRL> in red and <RLLR> in green) of cells with designated combinations of replichores affected by repressor-operator tight binding at 37°C. C. The SCA proportions of cells with designated combinations of replichores affected by repressor-operator tight binding at 30°C. D. The SCA proportions of L3*lacO*-R3*tetO* (‘LlRt’) or L3*tetO*-R3*lacO* (‘LtRl’) cells with designated combinations of replichores affected by repressor-operator tight binding at 37°C. E. Level and direction of changes in SCA proportions under the LacI-*lacO* tight binding condition when compared with corresponding ‘no tight binding’ reference values. The ‘37°C’ and ‘30°C’ columns are from Fig. 3B and C, showing the SCA proportion changes from ‘−’ to ‘L’ at 37°C and 30°C respectively. The ‘LlRt’ and ‘LtRl’ columns are from Fig. 3D, showing the SCA proportion changes from ‘−’ to ‘L′ and from ‘−’ to ‘R’, respectively, at 37°C.

New insight into this question came from an experiment originally aimed to characterize a L3 (*lacO)*-R3 *(tetO)* strain modified to have lower levels of LacI-CFP and TetR-YFP expression in order to minimize any aberrant effects due to repressor binding. To see how the new strain behaved when IPTG and/or AT were not present in the growth medium, we grew the strain in 30°C or 37°C medium containing four different combinations of IPTG and AT (Fig. 3A). The generation times of cells growing in different conditions were within a narrow range (125–135 min for 30°C and 110–120 min for 37°C), and the cell length distribution of these cell populations were practically identical (Fig. S1B and C). As LacI and/or TetR are expected to bind tightly to their respective operator arrays when IPTG and/or AT are absent, these results suggest that the cellular concentrations of either repressor were insufficient to maintain a high occupancy of binding to the operator arrays tight binding of LacI and/or TetR in the new strain, and thereby were ineffective in long-term blocking DNA replication, and consequent disruption of the cell cycle.

Repressor-operator tight binding, however, did affect the SCA proportions in a cell population. When both AT and IPTG were present, replication of neither replichore should be interrupted by tight repressor binding and the proportions of <LRLR>, <LRRL> and <RLLR> were in agreement with earlier data (Fig. 3B and C; Wang *et al*., 2006), irrespective of whether cells were grown at 37°C or 30°C. The proportion of <LRRL> was approximately equal to the proportion of <RLLR>. When only AT was present, potentially allowing transient pauses in replication at L3 (Fig. 3B and C; L), the proportion of <LRLR> showed highly statistically significant reductions at 37°C (74.5–66.7%; *P* < 0.05) and 30°C (74.3–66.5%; *P* < 0.05). These reductions were accommodated by a highly significant increase in the proportion of <RLLR> at 37°C (13.6–24.2%, *P* < 0.05) and 30°C (14.8–24.6%, *P* < 0.05). These observations support the hypothesis that transient pauses of replication on the left replichore at L3 by LacI-CFP binding, results in blocked sister loci being found towards the cell centre, as proposed (Wang *et al*., 2006).

When only IPTG was present, thereby potentially allowing transient blocks in replication at R3 (Fig. 3B and C; R), small reductions in the proportion of <LRLR> were observed at both temperatures (74.5–73.6% at 37°C and 74.3–73.5% at 30°C), but these reductions were not statistically significant. Small increases in <LRRL> at both temperatures were observed (11.9–14.8% at 37°C and 10.9–11.7% at 30°C, with the difference at 37°C being significant; *P* < 0.05).

When both inducers were absent, potentially allowing transient blocks in replication at both L3 and R3 (Fig. 3B and C; LR), a statistically significant reduction (*P*< 0.05) in <LRLR> was observed, with a concomitant statistically significant increase in <RLLR>, as if the blockage of the L replichore by LacI-CFP binding at L3 is greater than that by binding of TetR-YFP at R3, in agreement with the data where a single inducer is present.

In order to compensate for any unknown variables, we then swapped the *lacO* and the *tetO* arrays to generate an L3 (*tetO*)-R3 *(lacO*) strain and repeated the above experiments at 37°C using both the original and array-swapped strains, using the +AT +IPTG (no block) and +AT (block at R3 or L3) conditions. In the array-swapped strain, transient blocking at R3 increased the proportion of <LRRL> cells (17.5–23.2%, *P* < 0.05) and decreased the proportion of both <LRLR> and <RLLR> cells [71.2–68.5% (*P*< 0.06) and 11.3–8.3% (*P*< 0.05) respectively]. The control experiment using the original arrays showed the same trends as before (Fig. 3D).

Therefore, the effects of transient replication pausing by LacI-CFP binding to an array on the L, or R replichore, was the same (Fig. 3E). The proportion of cells in which the normal <LRLR> pattern is observed was reduced, while the mirror-symmetrical SCA in which the paused sister loci is found around midcell (<RLLR> for L3 replication pauses and <LRRL> for R pauses) was increased. The proportion of cells with the other mirror-symmetrical SCA (<LRRL> for L pauses and <RLLR> for R pauses) was also reduced. The effects of blocking at TetR-YFP-bound arrays showed the same trend, although the effects appear to be smaller, and not always statistically significant.

**Fig. 4:**
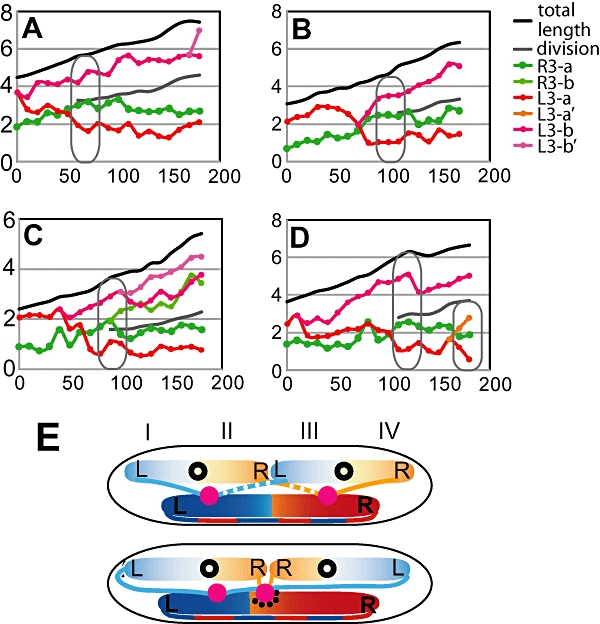
A–D. Time-lapse progressions showing L3-R3 loci after a replication block at R3. Replication block was applied to R3 (showed in green) by removing AT in M9 glycerol liquid culture for 60 min, while L3 (red and pink) could replicate as normal. The cells were then transferred to a slide mounted with the same medium plus 1% agarose. Pictures were taken every 10 min in a 3 h experiment. The schematics of cells (black cell-shaped outlines) are inserted to indicate the organization of loci in a cell of the indicated size. E. Schematic model for how replication directs chromosome organization. Replisomes are shown in pink. Leading and lagging strands are shown in smooth and dotted threads respectively. The replisome affected by pausing is marked with black dots. The whole cell is divided into four quarters marked by roman numbers. The upper cell adopts the *cis*-<LRLR> SCA, and the lower cell adopts the <LRRL> SCA.

To further test whether replication pausing or blocking influences chromosome organization, a complete replication block was applied to R3 by overexpressing TetR from the multicopy plasmid pWX6. A culture was blocked for 60 min in M9 glycerol liquid medium and an aliquot transferred to the same medium on an agarose pad for time-lapse analysis. Out of 121 cells analysed, 102 showed a similar behaviour; four examples are shown in Fig. 4A–D. In each case the blocked R3 locus (green) remains closes to the cell centre, prior to the formation of the septum and then proximal to the septum with respect to the sister L3 loci (red-orange) after cytokinesis is initiated. In one case (C), the ‘blocked’ locus eventually duplicates, generating the <LRRL> configuration, thereby strengthening the conclusion that replication blocks can generate the <LRRL> and <RLLR> sister chromosome configurations. The rare, yet informative, C class occurred in 12/121 cases analysed; these unambiguously generate the <LRRL> pattern that we postulate to result from the transient pausing experiments.

## Discussion

The data in this paper lead us to three main conclusions. First, we provide further data to support the hypothesis that the *E. coli* chromosome is normally organized as a sausage with a linker that connects the outer edges of the nucleoid, rather than as a doughnut shape in which chromosomal DNA is uniformly distributed around the ring. Although the precise amount of DNA in the linker is uncertain, it can be less than 42 kb, as loci separated by this distance can localize to opposite nucleoid edges. We do not know the precise organization of the linker, although loci that apparently fall in the middle of the linker region can appear as normal compacted foci, spatially separate from other *ter* markers (Fig. 1C).

Second, we show that at each cell cycle, the probability of generating each of the observed patterns of SCA is fixed and is independent of the SCA of the mother cell. A corollary of this is that within a population, each locus in a <LRLR> cell will segregate most often to the position occupied by the same locus in the mother cell, as if the position is imprinted. In contrast, segregation from a <LRRL> or <RLLR> cell will most often lead to one of the daughter loci being at a different cell position from its mother locus.

Finally, we provide experimental evidence that supports a direct mechanistic link between DNA replication and chromosome organization. Specifically, we show that site-specific pausing or blocking of replication of the R3 locus leads to the segregated L3 sister loci moving to the outer nucleoid edges and the blocked R3 locus remaining close to the midcell/invaginating septum. In situations where replication pausing is assumed to be transient, because the cell cycle is not significantly affected, we observe a switch from the normal <LRLR> configuration to the <RLLR> (L paused), or <LRRL> (R paused) configuration. Although we are unable to test whether replication arrest is responsible for the formation of endogenous <LRRL> and <RLLR> SCAs in exponentially growing populations, replication arrests requiring recombinational rescue are not involved because <LRRL> and <RLLR> frequencies are the same in wild type and *recA* strains (Wang *et al*., 2006).

The data here, when taken together with previous work (Wang *et al*., 2006; 2008; Reyes-Lamothe *et al*., 2008; White *et al*., 2008), lead to a model in which replication dynamics directly determines chromosome organization (Fig. 4E). After replication initiation, sister replisomes move quickly to opposite cell halves, thereby setting up the observed majority translation symmetry pattern, <LRLR>. This movement apart of sister replisomes may facilitate chromosome segregation. The mechanism that directs the two leading strand templates to the outer nucleoid edges (positions I and IV) remains unknown, but is presumably set up at, or soon after, replication initiation and may be important for the initial segregation process (White *et al*., 2008). Nevertheless, the segregation mechanism cannot demand that leading strand templates are directed to the outer nucleoid edges late in replication, because the <LRRL> type of configuration demands that both a leading- and lagging-strand template occupy positions I and IV at the outer nucleoid edges.

Once replication is well under way, it appears possible to remodel chromosome organization, as transient or long-term replication blockage ∼1600 kb away from *oriC,* when the chromosome is 70% replicated, allows an apparent switch from <LRLR> to <LRRL>, or <RLLR>, depending on whether the right or left chromosome arm, respectively, is replication-paused/blocked. Although our assays only monitor the change in position of L3/R3, and we do not know the position of other loci, we propose that the changes reflect changes of the whole sister chromosome. Nevertheless, we cannot eliminate the possibility that it is only the regions of the chromosome that are in the vicinity of the blocked locus that remain close to midcell. The arrangement of sister chromosomes may also change as a consequence of the wholesale nucleoid remodelling that goes on as newly replicated DNA replaces parental DNA in the growing nucleoid; sister replisomes and the associated chromosomal loci show dramatic positional changes over time, which are dependent on chromosome segregation as replication progresses (Reyes-Lamothe *et al*., 2008; Wang *et al*., 2008). These changes may generate the relatively rare switch from <LRLR> to <RLRL>. A future challenge is to track genetic loci and replisomes in relation to the changing positions and amounts of parental and newly replicated DNA.

## Experimental procedures

The *E. coli* AB1157 strains containing *lacO* and *tetO* L3-R3 arrays were constructed as described previously (Lau *et al*., 2003; Wang *et al*., 2006). *E. coli* AB1157 strains containing pMT1*parS* sites were constructed as in Nielsen *et al*. (2006b). *E. coli* MG1655 strains containing P1*parS* sites were used to prepare P1 lysates. To distinguish *parS*-marked loci, from array-containing loci, we use the nomenclature, L′ or R′ for *parS* loci. Conditions for bacterial growth and for visualizing fluorescent protein foci in living cells have been described (Wang *et al*., 2005, 2006). During exponential growth in M9 minimal glycerol medium, cells had a generation time of ∼100 min at 37°C, with non-overlapping sequential G1 (B), S (C) and G2/M (D) phases (Wang *et al*., 2005). Statistical tests of the significance of differences used the χ^2^ test.
