# Supplementary material for: Replication-directed sister chromosome alignment in Escherichia coli
Source: Mol Microbiol. 2009 Jul 24;75(5):1090–7. doi: 10.1111/j.1365-2958.2009.06791.x (PMC2859247; doi:10.1111/j.1365-2958.2009.06791.x)
Supplement: Supplementary file 1 [file mmi0075-1090-SD1.pdf]

Supplementary material: Liu et al

Calculation of 8-focus filament proportions (Fig. 2)

The proportions of the 10 type of 8-focus (4 chromosome) cephalexin filaments were calculated based on:

1) the lineage relationship that linked F0(mother), F1(daughter), and F2 (grand-daughter) chromosomes in cephalexin filaments according to scenario No.2,

2) the proportions of F0 chromosomes that adopted *cis*-<LRLR> (p_1), *trans*-<LRLR> (p_2), <LRRL> (p_3), <RLLR> (p_4) during the F0→F1 generation, and

3) the proportions of F1 chromosomes that adopted *cis*-<LRLR> (p_5), *trans*-<LRLR> (p_6), <LRRL> (p_7), <RLLR> (p_8) during the F1→F2 generation.

If p(<LRLR><LRLR>) was the proportion of <LRLR><LRLR> in a population of 8-focus cephalexin filaments, then we should have:

$$p(<LRLR><LRLR>) = (p_1+p_2)(p_5^2+p_6^2)+(p_3+p_4)p_5p_6$$

Similarly,

$p(<LRLR><RLRL>) = (p_1+p_2)p_5p_6+ p_3p_5^2+p_4p_6^2$

$p(<RLRL><LRLR>) = (p_1+p_2)p_5p_6 +p_3p_6^2+ p_4p_5^2$

$p(<LRLR><LRRL>) = (p_1+p_2)(p_5p_7+p_6p_7)+2p_3p_5p_7+ 2p_4p_6p_7$

$p(<LRLR><RLLR>) = (p_1+p_2)(p_5p_8+ p_6p_8)+2p_3p_5p_8+2p_4p_6p_8$

$p(<LRRL><LRLR>) = (p_1+p_2)(p_5p_7+p_6p_7)+ 2p_3p_6p_7+2p_4p_5p_7$

$p(<RLLR><LRLR>) = (p_1+p_2)(p_5p_8+p_6p_8)+2p_3p_6p_8+2p_4p_5p_8$

$p(<LRRL><LRRL>) = (p_1+p_2+p_3+p_4)p_7^2$

$p(<RLLR><RLLR>) = (p_1+p_2+p_3+p_4)p_8^2$

$p(<LRRL><RLLR>) = 2(p_1+p_2+p_3+p_4)p_7p_8$

The values of (p_1+p_2), p_3, and p_4, experimentally determined by examining F0→F1 events in the cell population before cephalexin treatment, were 75.22%, 10.78%, and 14.00%, respectively. The p_1/p_2 ratio was estimated to be 24.79 using time-lapse results shown in Fig 2D. Therefore, we have p_1=72.31%, p_2=2.92%, p_3=10.78%, p_4=14.00%.

The values of (p_5+p_6), p_7, and p_8, experimentally determined by examining the F1→F2 in 8-focus cephalexin filaments, were 77.87% , 9.51%, and 12.61%, respectively. Similarly, the p_5/p_6 ratio was estimated to be 24.79. Therefore we have p_5=74.86%, p_6=3.02%, p_7=9.51%, p_8=12.61%.

Using the formulas and the values of p_1-p_8, the predicted proportions of 10 types of 8-focus cephalexin filaments according to scenario No.2 were calculated and presented in Fig.2.

Fig S1. Cell size distributions

A) Cell length histograms of L1'-R1' and L2'-R2' cell populations which have been used to generate the data in Fig 1B. In Fig. 1B, only cells with 4 foci were selected for measurement. In Fig S1A, however, all the cells (with 2 or 4 foci) were measured.

B) and **C)** Cell length histograms of the cell populations which have been used to generate the data in Fig. 3 Cells growing at 30°C are shown in Fig S1B and cells growing at 37°C are shown in Fig S1C. In Fig. 3B and 3C, only cells with 4 foci were

selected for analysis. In Fig S1B or Fig S1C, however, all the cells (with 2 or 4 foci) were measured.

Fig S2. Cell micrographs

A) Two time-lapse series (20 min interval) of F0→F2 cell generations. The R3 foci are shown in red. In the upper panel, The F0→F1 generation is *cis*-<LRLR> and the two F1→F2 generations are *cis*-<LRLR> and <RLLR>. In the lower panel, the F0→F1 generation is <LRRL> and the two F1→F2 generations are *cis*-<LRLR> and *cis*-<LRLR>. **B)** Snapshot images of the 8-focus cephalexin filaments with the L3 foci shown in green and the R3 foci shown in red.

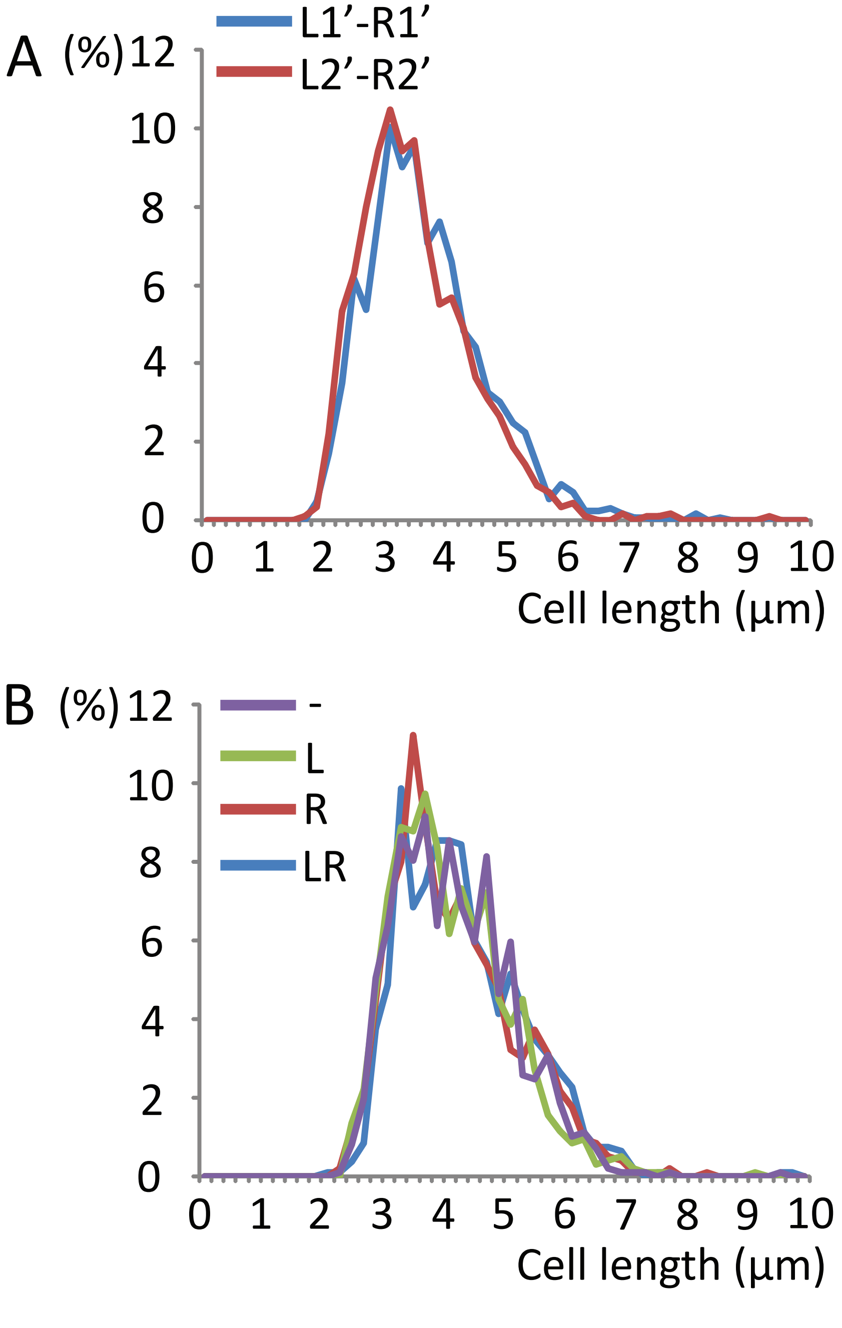

Fig S1_Liu et al.

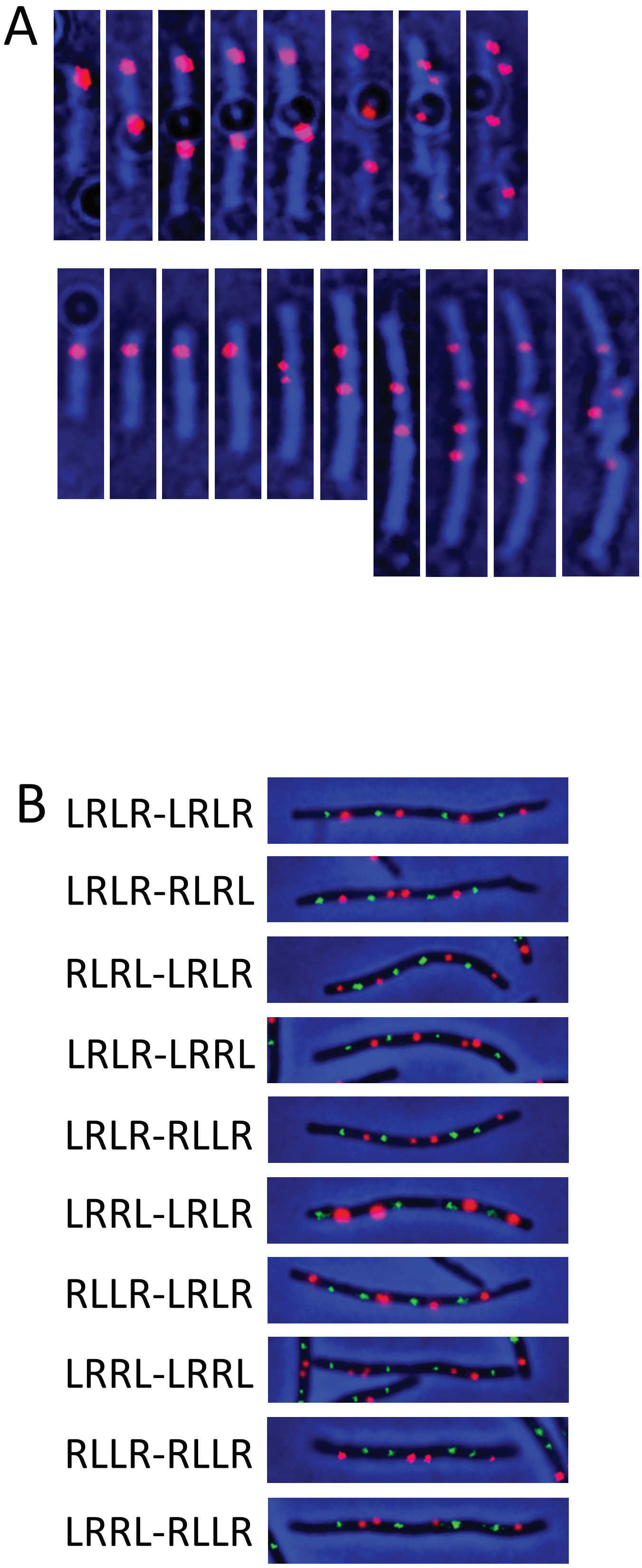

Fig. S2_Liu et al.
